# Metabolic indexes of obesity in major depressive disorders in different age groups

**DOI:** 10.1097/MD.0000000000047415

**Published:** 2026-01-30

**Authors:** Jieyu Zhou, Yukang Tan, Shasha Huang, Yajie Zhang, Jiaquan Liang

**Affiliations:** a Guangdong Provincial Hospital of Integrated Traditional Chinese and Western Medicine, Guangdong, People’s Republic of China; b Affiliated Guangdong Hospital of Integrated Traditional Chinese and Western Medicine of Guangzhou University of Chinese Medicine, Guangdong, People’s Republic of China; c Nanhai Hospital of Traditional Chinese Medicine, Jinan University, Guangdong, People’s Republic of China; d Nanhai District Hospital of Traditional Chinese Medicine of Foshan City, Guangdong, People’s Republic of China; e Department of Psychiatry, The Third People’s Hospital of Foshan, Guangdong, People’s Republic of China.

**Keywords:** BMI, major depressive disorder, metabolic indexes, obesity, overweight

## Abstract

Major depressive disorder (MDD) is a common chronic mental illness. Long-term treatment of MDD may lead to changes in internal factors, which may exacerbate obesity. Nevertheless, the metabolic abnormalities associated with MDD across various age groups remain poorly understood. To identify factors related to obesity in MDD patients of all ages during stable treatment, we collected 205 MDD patients divided into adolescent depression (10 ≤ age < 19), adult MDD (19 ≤ age < 60), and late-life depression (60 ≤ age), recorded their names, gender, and age, and then measured their weight, height, body mass index (BMI), and the metabolic indexes of obesity. Finally, the statistical analysis of these indicators was carried out to clarify the relationship between them. The results show that among MDD, with the increase of age, the risk of obesity increases. BMI was positively correlated with age, total cholesterol, triglyceride, low-density lipoprotein, aspartate aminotransferase, lactate dehydrogenase, uric acid, alanine aminotransferase, glycosylated hemoglobin, and high-sensitivity C-reactive protein, and negatively correlated with high-density lipoprotein. But creatine kinase isoenzyme, urea, serum creatinine, and homocysteine were not correlated with BMI. Multiple linear regression analysis showed that the value of BMI increased with the increase of age (*B* = 0.050, *P* = .002), aspartate aminotransferase (*B* = 0.094, *P* = .049), alanine aminotransferase (*B* = 0.055, *P* = .016), uric acid (*B* = 0.006, *P* < .021), glycosylated hemoglobin (*B* = 0.702, *P* = .004), and high-sensitivity C-reactive protein (*B* = 0.101, *P* < .001). In light of these findings, it is recommended that we should pay more attention to the monitoring of blood metabolism indicators with the increase of age in MDD, in order to facilitate timely clinical decision-making and reduce the risk of obesity in these individuals.

## 1. Introduction

Major depressive disorder (MDD) is a common chronic mental disorder characterized by continuous depression, lack of motivation or anhedonia, and loss of appetite. According to the China Mental Health Survey, its annual prevalence rate was about 4.1%.^[1]^ Regular and continuous use of drug treatment can help alleviate the condition of MDD patients, but long-term drug treatment (such as mirtazapine, paroxetine, etc) may lead to obesity, whether they are teenagers, adults, or the elderly.^[2–5]^ Previous studies^[6–9]^ and our previous research^[10,11]^ showed that after long-term medication, patients with mental illness such as MDD had abnormalities in biological factors, which might cause obesity, and increased the risk of other complications, such as circulatory system diseases,^[2]^ endocrine system diseases,^[12]^ and so on. Several studies have indicated that factors such as age, educational background, alcohol consumption, smoking habits, and lifestyle patterns are significantly associated with obesity.^[13]^ Additionally, in the context of blood metabolic indices, various parameters, including total cholesterol (TC),^[14]^ uric acid (UA),^[15]^ glycosylated hemoglobin (HbA1c),^[16]^ and low-density lipoprotein (LDL),^[17]^ have also been found to correlate with obesity. Nevertheless, the specific metabolic abnormalities associated with MDD across various age groups remain unclear. As clinicians, our aim is to refine these indicators and tailor monitoring strategies for patients with MDD in different age brackets.

Therefore, we collected blood samples from patients to identify the blood metabolism indicators that influence obesity in the context of MDD, intended to facilitate early intervention, minimize complications, enhance prognoses, and ultimately promote overall health.

## 2. Methods

### 2.1. Participants

Both outpatients and inpatients diagnosed with MDD were included in the study. Their diagnoses were confirmed by 2 psychiatrists using the Structured Clinical Interview for the Diagnostic and Statistical Manual of Mental Disorders, Fourth Edition^[18]^ from July 2016 to May 2023 in the Department of Psychiatry, The Third People’s Hospital of Foshan, Guangdong, People’s Republic of China, and the Guangdong Provincial Hospital of Integrated Traditional Chinese and Western Medicine. The participants were categorized into 3 age-based groups: adolescent depression (AD, 10 ≤ age < 19),^[19]^ adult MDD (19 ≤ age < 60), and late-life depression (LLD, 60 ≤ age),^[20]^ based on their respective age ranges. Meanwhile, they were required to maintain a fixed drug dose pattern for 2 years or more (in the stable period) before blood testing.

Exclusion criteria are as follows: comorbidity other mental disorders, including intellectual disability or other cognitive impairment; patients with diabetes and hypertension, severe, and unstable physical diseases, including severe kidney or liver function damage, cardiac insufficiency etc; those who did not cooperate with venous blood collection, such as phobia etc; smoking habits (≥1 cigarette per day); or drinking habits (≥1 unit alcohol per week): 1 unit alcohol = 480 to 600 mL of beer = 350 mL of low alcohol liquor or red wine, and yellow wine = 50 mL of high spirits (40° or more).

### 2.2. Assessments

To participate in the study, potential subjects who met the specified criteria were required to sign informed consent and partake in an interview to gather personal information, including name, gender, and age. Their physical dimensions, including height and weight, were measured by nurses, who computed the body mass index (BMI) for each participant.

To ensure accurate venous blood collection, subjects were instructed to fast for at least 8 hours before the procedure. The venipuncture was performed by nursing staff between 7:30 am and 10:00 am. Additionally, participants were advised to adhere to their regular dietary habits the night before the blood draw, maintain their usual work and sleep schedules, and avoid consuming alcohol or coffee after dinner.

The lipid profile, including TC, triglycerides (TG), high-density lipoprotein (HDL), and LDL, was assessed. Enzyme markers, such as aspartate aminotransferase (AST), lactate dehydrogenase (LDH), and creatine kinase, were evaluated. The creatine kinase isoenzyme (CK-MB), urea, serum creatinine (SCr), UA, and alanine aminotransferase (ALT) were also measured. Additionally, fasting blood glucose, HbA1c, high-sensitivity C-reactive protein (Hs-CRP), and homocysteine (Hcy) levels were documented for each subject in their clinical data records.

### 2.3. Statistics

The Kolmogorov–Smirnov test results for each measurement dataset across the groups indicated that all data adhered to a normal distribution and then utilized the Chi-square test to compare the count data across each group. The blood metabolic indexes of AD, adult MDD, and LLD were compared by 1-way analysis of variance, and then, Bonferroni post hoc analyses were performed. The relationships of BMI and variable indexes were analyzed by Pearson correlation, which was adjusted for age and gender. Next, the obtained *P*-values were then corrected by false discovery rate correction. Finally, multiple linear regression was utilized to examine the effects of blood index components on BMI.

### 2.4. Ethics

We obtained written informed consent from all patients or their legal guardians. This study received approval from the Ethics Committee of the Third People’s Hospital of Foshan, China, and all experiments were conducted in accordance with the principles outlined in the Declaration of Helsinki.

## 3. Results

### 3.1. Comparison of demographic characteristics and metabolic indexes in MDD of different age levels

There were 205 participants in this study, including AD (n = 57), adult MDD (n = 98), and LLD (n = 50), while 12 subjects were excluded because of eating breakfast before blood drawing or refusing blood test.

There were no significant differences in gender, height, weight, HDL, AST, ALT, LDH, CK-MB, and Hcy among AD, adult MDD, and LLD (*P* > .05). And there were significant differences in BMI, TC, TG, LDL, ALT, urea, SCr, UA, HbA1c, and Hs-CRP (*P* < .05). The incidence of obesity in AD, adult MDD, and LLD was 8.77%, 9.18%, and 14.28%, respectively. Overweight was 12.28%, 27.55%, and 46.43% (Table 1).

**Table 1:** Comparison of demographic characteristics and metabolic indexes.

	AD (n = 57)	Adult MDD (n = 98)	LLD (n = 50)	*F*	*P*
Age (yr)	16.07 ± 1.59	31.70 ± 9.98	64.75 ± 4.87	–	–
Gender (male/female)	17/40	32/66	21/29	1.94	0.379
Height (cm)	162.22 ± 8.02	163.02 ± 7.76	159.49 ± 8.26	2.17	0.117
Weight	56.84 ± 12.89	60.24 ± 11.72	63.08 ± 8.93	2.95	0.055
BMI (kg/m^2^)‡^,^§	21.48 ± 4.01	22.62 ± 3.80	24.79 ± 3.03	7.27	0.001*
Obesity (%)	8.77	9.18	14.28	–	–
Overweight (%)	12.28	27.55	46.43	–	–
Nonobesity (%)	78.95	63.27	39.29	–	–
TC (mmol/L)†^,^‡^,^§	4.60 ± 1.08	5.10 ± 1.09	5.55 ± 1.30	15.56	<0.001*
TG (mmol/L)‡^,^§	1.17 ± 0.80	1.31 ± 0.77	1.95 ± 1.00	19.61	<0.001*
HDL (mmol/L)	1.48 ± 0.33	1.51 ± 0.38	1.45 ± 0.47	0.26	0.772
LDL (mmol/L)†^,^§	2.46 ± 0.87	2.78 ± 0.84	3.05 ± 0.77	5.13	0.007*
AST (U/L)	18.79 ± 6.99	20.56 ± 10.27	22.14 ± 7.19	1.46	0.236
ALT (U/L)	19.56 ± 15.71	25.31 ± 23.12	25.64 ± 13.49	1.72	0.182
LDH (U/L)	167.02 ± 38.63	167.16 ± 33.73	178.32 ± 31.43	1.02	0.363
CK-MB (U/L)	13.00 ± 4.59	12.21 ± 3.55	13.98 ± 5.74	2.16	0.118
Urea (mmol/L)‡^,^§	4.23 ± 1.15	4.42 ± 1.17	5.30 ± 1.92	7.06	0.001*
SCr (μmol/L)†^,^‡^,^§	56.82 ± 11.20	63.85 ± 12.64	72.71 ± 25.02	11.55	<0.001*
UA (μmol/L)‡	346.07 ± 85.75	326.22 ± 97.62	391.46 ± 122.82	4.50	0.012*
HbA1c (%)‡^,^§	5.14 ± 0.33	5.30 ± 0.65	5.87 ± 1.09	10.89	<0.001*
Hs-CRP (mg/L)‡^,^§	1.89 ± 2.35	2.53 ± 5.17	3.89 ± 3.54	8.87	<0.001*
Hcy (μmol/L)	12.09 ± 9.48	11.32 ± 5.87	11.41 ± 5.22	0.21	0.807

Values are expressed as mean ± standard deviation.

AD = adolescent depression, adult MDD = adult major depressive depression, ALT = alanine aminotransferase, AST = aspartate aminotransferase, BMI = body mass index, CK-MB = creatine kinase isoenzyme, FDR = false discovery rate, HbA1c = glycosylated hemoglobin, Hcy = homocysteine, HDL = high-density lipoprotein, Hs-CRP = high-sensitivity C-reactive protein, LDH = lactate dehydrogenase, LDL = low- density lipoprotein, LLD = late-life depression, SCr = serum creatinine, TC = total cholesterol, TG = triglyceride, UA = uric acid.

* The comparison among groups (*P* < .05, 2-tailed, FDR correction).

† AD versus adult MDD.

‡ Adult MDD versus LLD.

§ AD versus LLD, *P* < .05.

### 3.2. Pearson correlation between BMI and various indexes in MDD

The results showed that BMI was positively correlated with age, TC, TG, LDL, AST, LDH, UA, ALT, HbA1c, and Hs-CRP, and negatively correlated with HDL. But CK-MB, urea, SCR, and Hcy were not correlated with BMI (Table 2).

**Table 2:** Pearson correlation between BMI and various indexes.

BMI								
Age*	*r*	0.389	AST*	*r*	0.172	SCr	*r*	0.114
	*P*	<.001		*P*	.020		*P*	.123
TC*	*r*	0.281	ALT*	*r*	0.328	UA*	*r*	0.335
	*P*	<.001		*P*	<.001		*P*	<.001
TG*	*r*	0.360	LDH*	*r*	0.251	HbA1c*	*r*	0.349
	*P*	<.001		*P*	.001		*P*	<.001
HDL*	*r*	−0.262	CK-MB	*r*	0.037	Hs-CRP*	*r*	0.244
	*P*	<.001		*P*	.620		*P*	.001
LDL*	*r*	0.369	Urea	*r*	0.086	Hcy	*r*	0.013
	*P*	<.001		*P*	.245		*P*	.862

ALT = alanine aminotransferase, AST = aspartate aminotransferase, BMI = body mass index, CK-MB = creatine kinase isozyme, FDR = false discovery rate, HbA1c = glycosylated hemoglobin, Hcy = homocysteine, HDL = high-density lipoprotein, Hs-CRP = high-sensitivity C-reactive protein, LDH = lactate dehydrogenase, LDL = low-density lipoprotein, SCr = serum creatinine, TC = total cholesterol, TG = triglyceride, UA = uric acid.

* *P* < .05, 2-tailed, FDR correction.

### 3.3. Multiple linear regression analysis of the influencing factors of BMI in MDD

Taking BMI as a dependent variable (*Y*), age, TC, TG, HDL, LDL, AST, ALT, LDH, UA, HbA1c, and Hs-CRP as independent variables (X), and gender as the covariate, a stepwise multiple linear regression model (*F* = 9.897, *P* < .001) was established. Finally, the elements entering the model were age, AST, ALT, and UA (Table 3).

**Table 3:** Multiple linear regression analysis of influencing factors of BMI.

Model	*B*	Standard error	Standard coefficient	*t*	*P*	95% CI	
(Constant)	13.468	2.603	–	5.175	<.001*	8.331	18.606
Age	0.050	0.016	0.223	3.138	.002*	0.019	0.081
TC	−0.991	1.246	−0.295	−0.795	.428	−3.450	1.468
TG	0.493	0.385	0.108	1.281	.202	−0.267	1.253
HDL	−0.151	1.497	−0.015	−0.101	.920	−3.105	2.803
LDL	1.981	1.537	0.438	1.288	.199	−1.054	5.016
AST	−0.094	0.049	−0.217	−1.945	.049*	−0.190	0.001
ALT	0.055	0.023	0.283	2.441	.016*	0.011	0.100
LDH	0.009	0.008	0.085	1.243	.216	−0.006	0.024
UA	0.006	0.003	0.161	2.325	.021*	0.001	0.012
HbA1c	0.646	0.391	0.116	1.651	.101	−0.126	1.419
Hs-CRP	0.037	0.060	0.040	0.613	.541	−0.081	0.155

ALT = alanine aminotransferase, AST = aspartate aminotransferase, BMI = body mass index, HbA1c = glycosylated hemoglobin, HDL = high-density lipoprotein, Hs-CRP = high-sensitivity C-reactive protein, LDH = lactate dehydrogenase, LDL = low-density lipoprotein, TC = total cholesterol, TG = triglyceride, UA = uric acid.

## 4. Discussion

Our study included AD, adult MDD, and LLD who had treated with fixed doses of drugs for 2 years or more, and excluded smoking and drinking habits. The indicators we measured contained blood lipid, liver function, renal function, cardiac function, blood inflammation, and so on, which could comprehensively understand the related factors between MDD and obesity.

When comparing MDD across different age groups, we observed minimal differences in the incidence of obesity between adolescents and adults, while the incidence of obesity was notably higher in patients with LLD. According to a national epidemiological survey, the results indicated that the prevalence of obesity in urban areas in southern China was 2.8% to 7.2% from 2010 to 2018,^[21]^ where the subjects in our study came from. This means that the incidence of obesity in patients with MDD treated regularly would increase at different ages.

Additionally, the values of TC, TG, LDL, urea, SCr, UA, HbA1c, and Hs-CRP exhibited an upward trend in the LLD. To further investigate the relationship between these serum metabolic indices and the occurrence of obesity in MDD, we conducted a correlation analysis between BMI and these parameters. The results indicated that BMI was positively correlated with age, TC, TG, LDL, AST, ALT, LDH, UA, HbA1c, and Hs-CRP, while it was negatively correlated with HDL. Previous research findings have indicated that the incidence of obesity tends to increase with age, and this pattern was also observed in cases of MDD.^[22]^ TC, TG, and LDL are the most commonly measured blood lipid indicators in clinical settings and are widely recognized as risk factors in lipid metabolism.^[10,23,24]^ Therefore, it is easy to understand that their rise increases the risk of obesity. In contrast, HDL has the opposite function and its rose was a protective factor for obesity.^[25,26]^ These were consistent with our experimental results. Clinically, AST and ALT are commonly used to reflect human liver function, and the liver function plays an important role in lipid metabolism.^[27]^ LDH is primarily found in animal tissues such as the myocardium and liver. Clinically, it serves as an auxiliary index for diagnosing myocardial infarction and liver diseases. Similar to AST and ALT, elevated LDH levels are implicated in the pathophysiological processes of obesity and overweight.^[28]^ SCr is a byproduct of human muscle metabolism. In muscle tissue, creatine undergoes a slow, irreversible nonenzymatic dehydration reaction to form creatinine, which is subsequently released into the bloodstream, filtered by the glomerulus, and excreted in urine. The measurement of SCr serves as a crucial indicator of renal function, with elevated levels signifying impaired kidney function. In longitudinal studies on diabetes, researchers have observed that obesity is associated with a decline in renal function.^[29]^ The results of this study indicated that there were significant differences in the comparison of SCr in different age groups, and SCr in the older group is higher. However, when we did correlation analysis between SCR and BMI, we got negative results, which were inconsistent with our previous results^[10]^ and might be the impact of the selection of MDD in this study. The impact of UA is well-documented, with some studies suggesting that elevated UA levels are linked to weight gain and hypertension. And the increase in UA is significantly associated with a heightened risk of diabetes, particularly in overweight individuals.^[30,31]^ HbA1c is the reflection of the average blood glucose level in the body for 3 months. It was thought to be a risk factor for obesity and diabetes.^[32–34]^ Hs-CRP is an acute-phase protein synthesized in the process of inflammatory stimulation, such as tissue injury and infection. As a member of inflammatory factors, it participated in the disease process of MDD^[35]^ and was considered to be related to obesity.^[36]^ Therefore, our study verified the previous research and achieved good consistency.

The results of regression analysis showed that age, AST, ALT, and UA were the risk factors for the increase in BMI. To our surprise, conventional blood lipid indexes, such as TC, TG, LDL, and HDL, did not pass the test. As we know, antidepressants are metabolized by liver and kidney, and taking such drugs will often affect liver and kidney function clinically, especially for ALT, AST, UA, etc.^[37–39]^ Our results were the same, suggesting that the increase of these indicators is of great significance in the occurrence of obesity in MDD in different age groups.

However, this study does have its limitations. The participants were predominantly from southern China, which may not fully represent the broader population. Therefore, the generalization of our findings should be approached with caution.

In conclusion, we should pay more attention to the monitoring of blood metabolism indicators with the increase of age in MDD, in order to facilitate timely clinical decision-making and reduce the risk of obesity in these individuals.

## Author contributions

**Data curation:** Yukang Tan.

**Formal analysis:** Yukang Tan.

**Funding acquisition:** Jiaquan Liang.

**Investigation:** Shasha Huang, Yajie Zhang.

**Methodology:** Jiaquan Liang.

**Project administration:** Shasha Huang.

**Resources:** Yajie Zhang, Jiaquan Liang.

**Software:** Yajie Zhang, Jiaquan Liang.

**Supervision:** Jiaquan Liang.

**Validation:** Jiaquan Liang.

**Writing – original draft:** Jieyu Zhou, Jiaquan Liang.

**Writing – review & editing:** Jieyu Zhou, Jiaquan Liang.
